# Quantitative Real-Time PCR Analysis of YKL-40 and Its Comparison with Mammalian Chitinase mRNAs in Normal Human Tissues Using a Single Standard DNA

**DOI:** 10.3390/ijms16059922

**Published:** 2015-04-30

**Authors:** Misa Ohno, Peter O. Bauer, Yuta Kida, Masayoshi Sakaguchi, Yasusato Sugahara, Fumitaka Oyama

**Affiliations:** 1 Department of Chemistry and Life Science, Kogakuin University, Hachioji, Tokyo 192-0015, Japan; E-Mails: bd13001@ns.kogakuin.ac.jp (M.O.); yuta.maldini@blue.email.ne.jp (Y.K.); bt11532@ns.kogakuin.ac.jp (M.S.); bt79310@ns.kogakuin.ac.jp (Y.S.); 2 Research Fellow of Japan Society for the Promotion of Science (DC2), Koujimachi, Chiyoda-ku, Tokyo 102-0083, Japan; 3 Department of Neuroscience, Mayo Clinic, Jacksonville, FL 32224, USA; E-Mail: bauer.peter@mayo.edu

**Keywords:** asthma, chitinase, chitinase-like protein, cystic fibrosis, gene expression analysis, malignant tumors, normal human tissues, quantitative real-time PCR system, rheumatoid arthritis, YKL-40

## Abstract

YKL-40 (YKL for the first three *N*-terminal residues of a 40 kDa protein) belongs to a group of human chitinase-like proteins (CLPs), which are similar to chitinases but lack chitinolytic activity. YKL-40 mRNA and its protein levels have been reported elevated in multiple disorders including asthma, cystic fibrosis, rheumatoid arthritis and malignant tumors. Here, we quantified the YKL-40 mRNA levels and compared them with chitinases and housekeeping genes in normal human tissues. To establish the quantitative real-time PCR (qPCR) system for evaluation of relative YKL-40 mRNA levels, we constructed a human standard DNA molecule by ligating cDNAs of YKL-40, two mammalian chitinases and two housekeeping genes in a one-to-one ratio. We generated cDNAs from various normal human tissues and analyzed the YKL-40 mRNA expression levels using a qPCR system with the standard DNA. We found that YKL-40 mRNA is present widely in human tissues while its expression patterns exhibit clear tissue specificity. Highest YKL-40 mRNA levels were detected in the liver, followed by kidney, trachea and lung. The levels of YKL-40 mRNA in the kidney and liver were more than 100-times higher than those of chitotriosidase mRNA. Our study provides for the first time a comprehensive analysis of the relative expression levels of YKL-40 mRNA *versus* mammalian chitinases in normal human tissues.

## 1. Introduction

Chitinases are enzymes that digest chitin, a polymer of (β-1-4)-linked *N*-acetyl-d-glucosamine (GlcNAc) and an integral component of the crustaceans and insect exoskeletons, microfilarial sheath of parasitic nematodes and fungal cell walls [1,2]. In humans and mice, active chitinases are encoded by two genes, chitotriosidase (Chit1) and acidic mammalian chitinase (AMCase) [2,3,4]. Chit1 is an active and well-characterized chitinase and it is the first mammalian chitinase that was purified and cloned [5,6,7]. AMCase was discovered later and gained its name due to its acidic isoelectric point [8,9].

Family 18 of the glycosyl hydrolases contains mammalian chitinases and chitinase-like proteins (CLPs) based on the primary structure similarities [4,10,11,12]. The conserved sequence in family 18 of the chitinases (DXXDXDXE) is involved in catalysis and the glutamic acid (E) amino acid is assumed to be the catalytic residue [4,10,13].

CLPs share structural homology with chitinases but lack the ability to degrade chitin. Multiple CLPs have been identified in humans and mice [14,15,16,17,18,19,20,21,22]. Humans produce primarily YKL-40 (CHI3L1 or human cartilage glycoprotein-39), whereas mice mainly express breast regression protein-39 (BRP-39) (chitinase 3-like-1 (Chi3l1) or 38-kDa glycoprotein (gp38k)), mouse homologue of YKL-40, Ym1 (Chi3l3) and Ym2 (Chi3l4). In humans, Ym1 and Ym2 are not present. It is generally accepted that CLPs lack chitinase activity due to evolutionary mutations of crucial residues within the conserved catalytic domain [4,10,13].

YKL-40 is secreted as a 40-kDa glycoprotein from various cell types, which include macrophages, chondrocytes and tumor cells [14,18,23,24]. The amino acid sequence of YKL-40 shares 73% identity with BRP-39 [23,24] and a recent study has shown that these proteins are functionally equivalent [25]. Recently, YKL-40 has attracted considerable attention due to its increased levels in individuals with asthma, chronic obstructive pulmonary disease (COPD), cystic fibrosis, rheumatoid arthritis, inflammatory bowel disease, alcoholic cirrhosis and different types of malignant tumors [26,27,28,29,30,31,32,33,34,35,36,37,38].

Despite lacking chitinase activity, YKL-40 has been implicated in multiple inflammatory diseases [39]. In accordance, recent studies have reported association between expression levels of two mammalian chitinases and inflammatory conditions [5,40,41,42,43,44]. Quantification and comparison of YKL-40 with mammalian chitinases and housekeeping genes are important steps toward understanding the *in vivo* regulation of YKL-40.

Recently, we established a quantitative real-time PCR (qPCR) system using a single standard DNA molecule to quantify the expression levels of CLPs, chitinases and reference genes [45,46,47]. This tool enables us to analyze the expression levels of multiple genes at the same scale. In this study, we applied our qPCR system for quantification of the YKL-40 expression while comparing its levels to those of chitinases and reference genes in human tissues. We found that YKL-40 is highly expressed in several tissues including liver, kidney, trachea, and lung. The levels of YKL-40 mRNA in the kidney and liver were more than 100-times higher than those of Chit1 mRNA, although they were lower than the levels of glyceraldehyde-3-phosphate dehydrogenase (GAPDH) and β-actin [48,49,50].

## 2. Results

### 2.1. Establishment of the Real-Time PCR System for Detection of YKL-40 mRNA in Human Tissues

**Figure 1:**
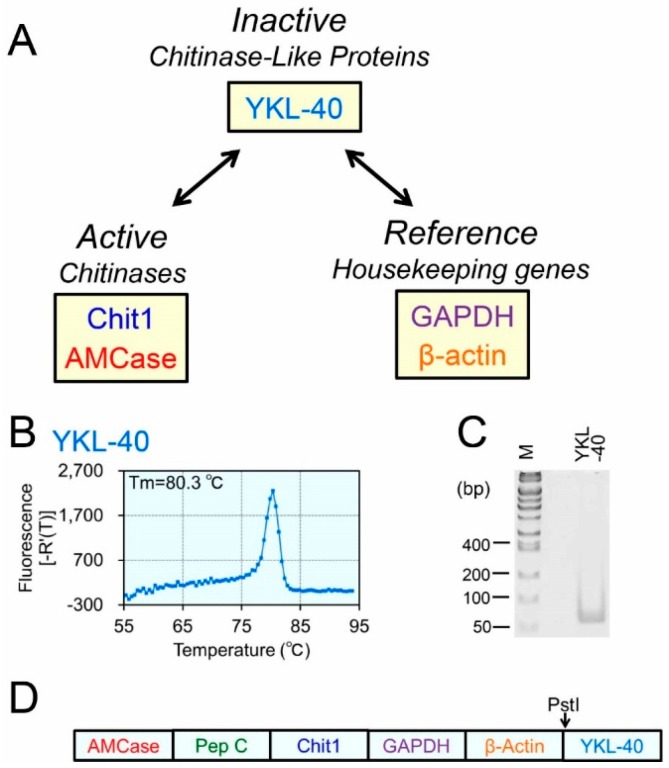
Strategy for comparing the gene expression levels of YKL-40, chitinases and housekeeping genes and construction of the Refs/YKL-40 standard DNA. (**A**) The expression of YKL-40 was quantified and subsequently, the levels of YKL-40, active chitinases (Chit1 and AMCase) and housekeeping genes (GAPDH and β-actin) were compared; (**B**,**C**) Evaluation of primer suitability for qPCR. The primers were evaluated based on whether they displayed single melting temperature (**B**) and a single PCR product on a 10% polyacrylamide gel (**C**); The *y*-axis was expressed as first derivative of the fluorescence as a function of temperature (**B**). To verify the specificity, the dissociation curves of YKL-40 PCR products were generated using a human tissue cDNA mixture. The PCR products were analyzed polyacrylamide gel electrophoresis, followed by ethidium bromide staining; and (**D**) schematic representation of the human Refs/YKL-40 standard DNA used for real-time PCR analysis.

We previously established a real-time PCR system that is capable of quantifying mRNA expression of two mammalian chitinases in mouse tissues while comparing their levels with those of reference genes using the same scale [45,46,47]. In this study, we aimed to quantify the levels of YKL-40 (inactive protein) and compare them with expression of Chit1, AMCase (active chitinases) and housekeeping genes GAPDH and β-actin in human tissues (Figure 1A).

We first designed primers to quantify YKL-40 (Figure S1) and evaluated their suitability based on a single melting temperature (Tm) and a single band on a 10% polyacrylamide gel (Figure 1B,C). We confirmed that the YKL-40 fragment was efficiently amplified from the human tissue cDNA mixture using the YKL-40 primers (Figure S1).

### 2.2. Construction of the Human Refs/YKL-40 Standard DNA and Validation of Our qPCR System

**Figure 2:**
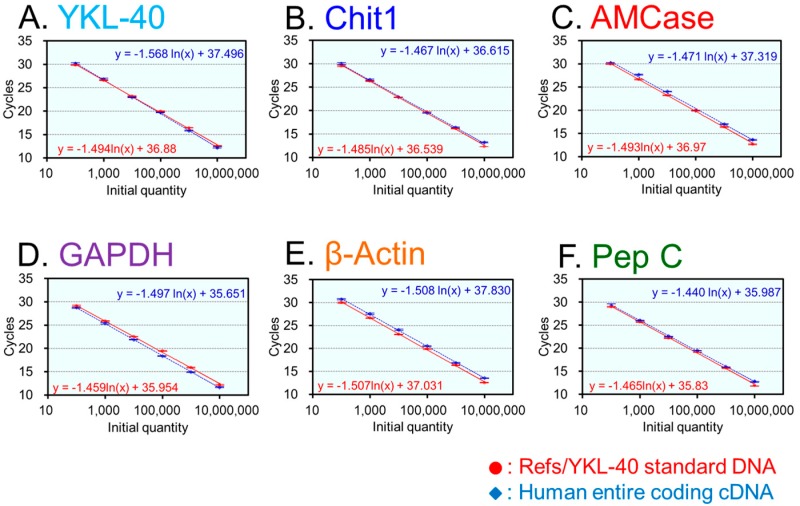
Validation of the qPCR system for the human tissues analysis. Following cDNAs were analyzed: (**A**) YKL-40; (**B**) Chit1; (**C**) AMCase; (**D**) GAPDH; (**E**) β-actin; and (**F**) pepsinogen C. Standard curves were obtained using the Refs/YKL-40 standard DNA containing six human cDNA fragments (red closed circles). In addition, the quantification of the human entire coding cDNA was performed using specific primer pairs for each gene. Target cDNA was amplified from a diluted sample of entire coding cDNA with a known concentration and subsequently analyzed as an unknown sample (blue closed rhombuses). Equal quantities were obtained for each tested dilution of the standard curve and entire coding cDNA. Data are expressed as mean ± standard deviation (SD) of three independent measurements.

Quantification of YKL-40, chitinases and the reference mRNAs relies on well-constructed standard curves. We examined whether YKL-40 and five reference mRNAs were quantified accurately using this system. With serial dilutions of the human Refs/YKL-40 standard DNA (Figure 1D and Figure S2), we constructed individual standard curves to evaluate the qPCR strategies analyzing six tested mRNAs. Each standard curve was generated using 10-fold serial dilutions of the standard DNA and six primer pairs, yielding a dynamic range of seven orders of magnitude (Figures 2A–F, red closed circles).

We next validated our qPCR system by analyzing six cDNA targets (Figure 2). To test the absolute equality of the curves, known concentration of the entire coding cDNA (Figure S3) was amplified and subsequently analyzed as an unknown sample. As shown in Figure 2A–F, blue closed rhombuses, equal quantities were observed for each tested dilution used to construct the standard curve. Thus, we were able to quantify YKL-40 and the reference mRNAs using the same scale.

### 2.3. Expression Levels of the YKL-40 mRNA in Normal Human Tissues

**Figure 3:**
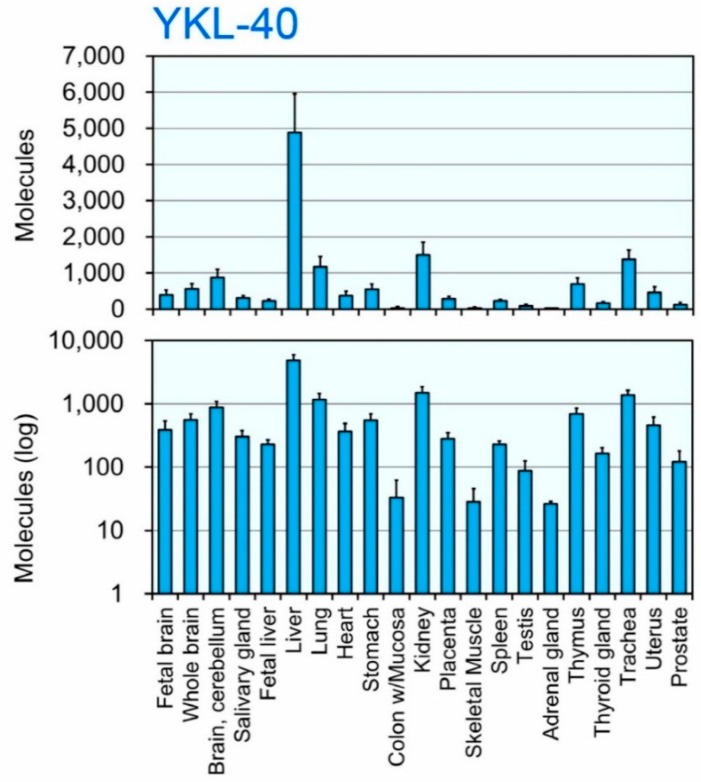
The expression of YKL-40 mRNA in normal human tissues. The expression levels of YKL-40 were quantified by qPCR using human Refs/YKL-40 standard DNA. The *y*-axis represents molecules per 10 ng of total RNA. **Upper panel** indicates the actual value and the **lower panel** shows the logarithm of each value. Human tissue samples were pooled from 1–64 Caucasians: fetal brain, *n* = 59; whole brain, *n* = 1; cerebellum, *n* = 10; salivary gland, *n* = 24; fetal liver, *n* = 63; liver, *n* = 1; lung, *n* = 3; heart, *n* = 3; stomach, *n* = 1; colon, *n* = 1; kidney, *n* = 1; placenta, *n* = 15; skeletal muscle, *n* = 2; spleen, *n* = 12; testis, *n* = 39; adrenal gland, *n* = 62; thymus, *n* = 2; thyroid gland, *n* = 64; trachea, *n* = 22; uterus, *n* = 8; prostate, *n* = 12.

To study the *in vivo* regulation of YKL-40 gene expression, total RNA from various normal human tissues was analyzed using our qPCR assay in the presence of the human Refs/YKL-40 standard DNA (Figure 1D and Figure S2). Human tissue samples were pooled from 1–64 Caucasians: fetal brain, *n* = 59; whole brain, *n* = 1; cerebellum, *n* = 10; salivary gland, *n* = 24; fetal liver, *n* = 63; liver, *n* = 1; lung, *n* = 3; heart, *n* = 3; stomach, *n* = 1; colon, *n* = 1; kidney, *n* = 1; placenta, *n* = 15; skeletal muscle, *n* = 2; spleen, *n* = 12; testis, *n* = 39; adrenal gland, *n* = 62; thymus, *n* = 2; thyroid gland, *n* = 64; trachea, *n* = 22; uterus, *n* = 8; prostate, *n* = 12. In Figure 3, upper panel indicates the actual value and the lower panel shows the logarithm of each value. We found that YKL-40 mRNA is widely expressed throughout human tissues (Figure 3) with highest levels detected in the liver, followed by kidney, trachea and lung tissues (Figure 3, upper panel). In the rest of the tissues, YKL-40 mRNA was expressed at low, but detectable levels well above the background (Figure 3, lower panel). We confirmed that no amplicons were produced from control solution in the absence of cDNA with qPCR primers for YKL-40. Therefore, we were able to quantify the expression levels of the middle point between 0 and 100 (in log scale).

### 2.4. Comparison of YKL-40, Mammalian Chitinases and Housekeeping Genes mRNA Levels in Healthy Human Tissues

**Figure 4:**
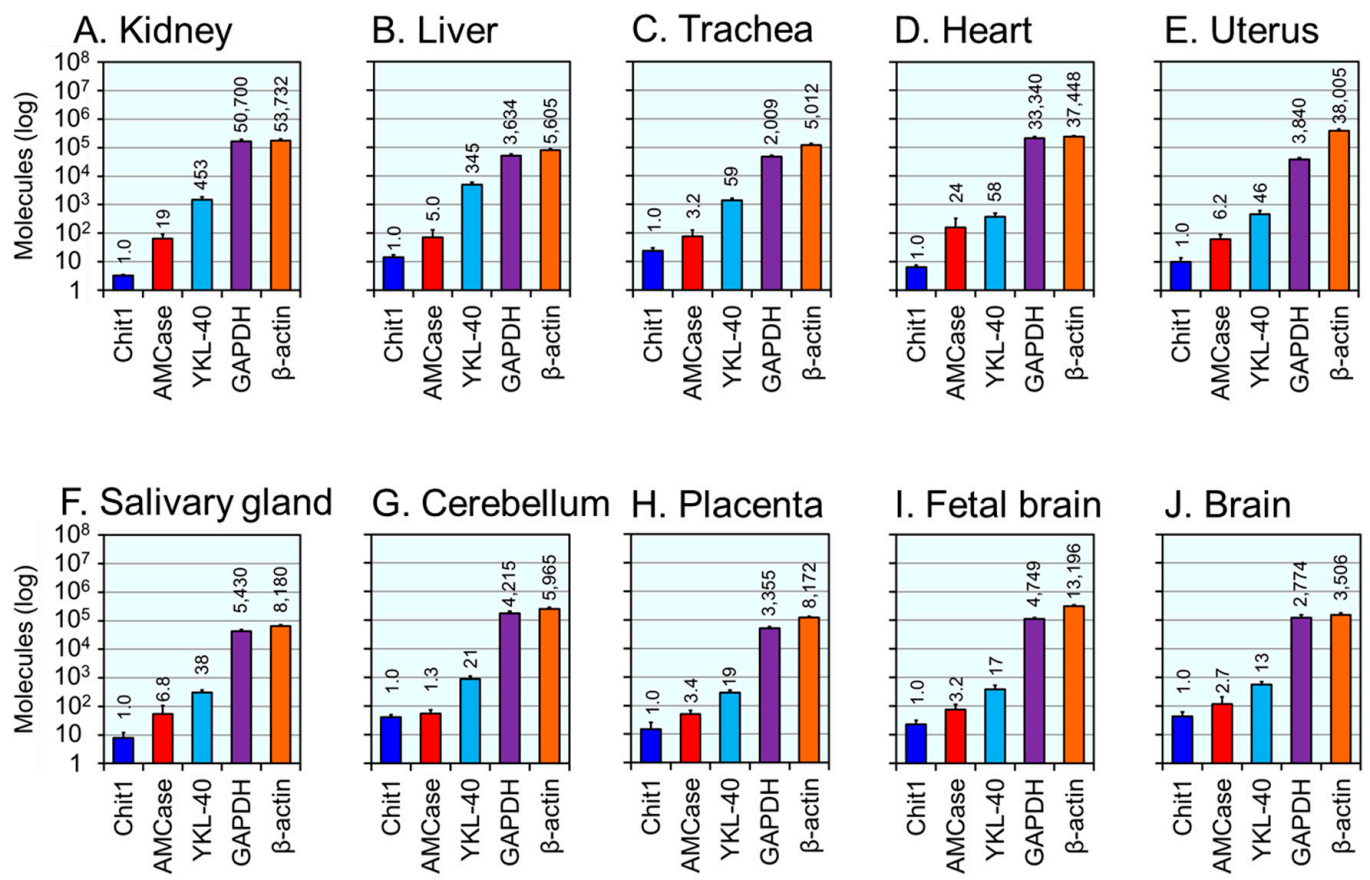
Human tissues with expression levels of YKL-40 more than 10-times higher than those of Chit1. Expression analysis of five genes in kidney (**A**); liver (**B**); trachea (**C**); heart (**D**); uterus (**E**); salivary gland (**F**); cerebellum (**G**); placenta (**H**); fetal liver (**I**) and whole brain (**J**) tissues using qPCR. All values are expressed as number of molecules per 10 ng of total RNA in *y*-axis. Logarithm of each value is shown. The expression level of Chit1 was set to 1.0; the values above bars indicate the relative expression levels compared to Chit1. Human tissue samples were pooled from 1–64 Caucasians: kidney, *n* = 1; liver, *n* = 1; trachea, *n* = 22; heart, *n* = 3; uterus, *n* = 8; salivary gland, *n* = 24; cerebellum, *n* = 10; placenta, *n* = 15; fetal brain, *n* = 59; whole brain, *n* = 1.

**Figure 5:**
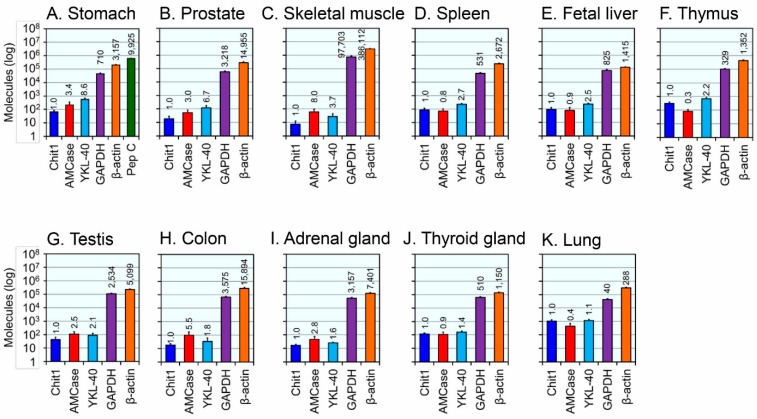
Human tissues with expression levels of YKL-40 similar or less than 10-times higher than those of Chit1. Expression analysis of six genes in stomach (**A**) and five genes in prostate (**B**); skeletal muscle (**C**); spleen (**D**); fetal liver (**E**); thymus (**F**); testis (**G**); colon (**H**); adrenal gland (**I**); thyroid gland (**J**) and lung (**K**) tissues using qPCR. All values are expressed as number of molecules per 10 ng of total RNA in y axis. Logarithm of each value is shown. The expression level of Chit1 was set to 1.0; the values above bars indicate the relative expression levels compared to Chit1. Human tissue samples were pooled from 1–64 Caucasians: stomach, *n* = 1; prostate, *n* = 12; skeletal muscle, *n* = 2; spleen, *n* = 12; fetal liver, *n* = 63; thymus, *n* = 2; testis, *n* = 39; colon, *n* = 1; adrenal gland, *n* = 62; thyroid gland, *n* = 64; lung, *n* = 3.

We next compared the expression levels of YKL40, Chit1, AMCase, GADPH and β-actin in healthy human tissues (Figure 4 and Figure 5).

Figure 4 shows ten human tissues where YKL-40 mRNA level was more than 10-times higher than the Chit1 level. With Chit1 levels set at 1.0, the relative expression levels of YKL-40 was 453 in kidney, 345 in liver, 59 in trachea, 58 in heart, 46 in uterus, 38 in salivary gland, 21 in cerebellum, 19 in placenta, 17 in fetal brain and 13 in brain (Figure 4). The expression of YKL-40 predominated over the active chitinases in these tissues with levels being more than 100-times higher in the kidney and liver as compared to Chit1 (Figure 4A,B). On the other hand, the YKL-40 levels were markedly lower than those of the housekeeping genes (Figure 4A,B).

In Figure 5, 11 tissues with YKL-40 mRNA levels less than 10-times higher or comparable to Chit1 are shown. When compared to Chit1, relative expression levels of the YKL-40 were 8.6 in stomach, 6.7 in prostate, 3.7 in skeletal muscle, 2.7 in spleen, 2.5 in fetal brain, 2.2 in thymus, 2.1 in testis, 1.8 in colon, 1.6 in adrenal gland, 1.4 in thyroid and 1.1 in lung (Figure 5). Skeletal muscle, testis, colon and adrenal gland tissues expressed more AMCase than YKL-40 (Figure 5C,G,H,I). In the lung, the expression level of YKL-40 was identical to the level of Chit1 (Figure 5K).

## 3. Discussion

In this study, we quantified the expression of a chitinase-like protein (CLP), YKL-40 and compared its mRNA levels with those of the active mammalian chitinases and housekeeping genes in healthy human tissues. We also revealed the tissue-specific YKL-40 expression pattern.

In our previous studies, we quantified and compared Chit1 and AMCase expression levels in mouse and human tissues [45,46]. Additionally, we applied our methodology to analyze the CLPs’ levels in mouse tissues [47]. To prevent amplification of off-target fragments by cross-reaction of the Ym2 primers, we prepared a linearized DNA molecule containing pGEM-T Easy sequence situated between the Ym1 and Ym2 cDNA, serving as an intron-like insertion and changed our qPCR protocol (annealing, 30 s at 55 °C; extension, 10 s at 72 °C) [47]. With these modifications, we improved our technique and after the validation of the mouse Refs/CLPs standard DNA with pGEM-T Easy, we were able to individually quantify Ym1 and Ym2 [47]. Consequently, we applied our methodology for YKL-40 levels analysis in human tissues. Since no cross-reaction of the YKL-40 primers with the human Refs/CLPs standard DNA occurred, we used the Refs/YKL-40 standard DNA without the vector sequence insertion.

Our analysis was sufficiently sensitive to detect YKL-40 and to provide a comprehensive survey of the gene expression patterns of YKL-40, two active chitinases and two reference genes using the same scale in human tissues. YKL-40 expression was highest in the human liver tissue followed by kidney (Figure 3). As the liver and kidney RNA samples were obtained only from one individual, this data should be confirmed in a larger sample number. The expression levels of BRP-39 in the mouse lung were higher than those of two active chitinases and were comparable to GAPDH [47]. In contrast, YKL-40, the human homolog of BRP-39, was not overexpressed in the human lung (Figure 3). Thus, the expression level of BRP-39/YKL-40 in lung tissues appears to be species-specific. A detailed characterization of the *cis-* and *trans*-acting factors will be required to understand the selective gene expression of this gene in humans.

In this study, we revealed expression levels ratios of YKL-40, two mammalian chitinases and two housekeeping genes in 21 human tissues (Figure 4 and Figure 5). The expression of YKL-40 was higher in all tested tissues except for lung as compared to Chit1 (Figure 4 and Figure 5). Numerous studies have shown elevated YKL-40 expression in inflammatory diseases [26,27,28,29,30,31,32,33,34,35,36,37,38] and it has been suggested that it may be associated with tissue remodeling [24,33]. Hence, the importance of YKL-40 and other CLPs in tissue defense seems to be independent on chitinolytic activity. Our data can help to understand the biological function of YKL-40 and chitinases, particularly in future pathophysiological studies.

Besides YKL-40, increased chitinases levels have also been observed under inflammatory conditions [26,27,28,29,30,31,32,33,34,35,36,37,38]. The Chit1 level is elevated in Gaucher disease, in smokers and in patients with COPD and Alzheimer disease [5,40,41,43]. AMCase expression and activity are up-regulated during allergic airway responses in mouse models of asthma and after polymeric chitin administration [51,52]. Thus, chitinases and CLPs may play important roles in different pathophysiological conditions [2,3,10], however the individual contributions of these proteins remain to be determined.

Using BRP-39-deficient and YKL-40 transgenic mice, it has been demonstrated that these proteins are functionally equivalent and play similar roles in tissue remodeling, regulation of the cell death pathway and airway obstruction [25]. Moreover, BRP-39 is induced upon bacterial infection, promoting bacterial clearance by controlling cell death, inflammation and remodeling via interleukin (IL)-13 receptor α2 [53,54]. The quantification system presented in this study can be used to analyze YKL-40 mRNA levels in tissues affected by different pathological processes, including those described above and help to understand the biological functions of chitinases and CLPs in pathophysiological studies.

## 4. Experimental Section

### 4.1. RNA and cDNA Preparation

The qPCR assay has been designed according to the Minimum Information for Publication of Quantitative Real-Time PCR Experiments (MIQE) guidelines [55,56].

We used the commercially available Human Total RNA Master Panel II (Clontech Laboratories, Mountain View, CA, USA) and Human Stomach Total RNA (Clontech Laboratories). We used them to examine the distribution of the transcripts in various human tissues. The samples of total RNA (3 µg) were subjected to reverse transcription using random hexamers. The reaction mixture (15 µL) contained enzyme buffer (50 mM Tris-HCl (pH 8.3), 75 mM KCl and 3 mM MgCl_2_), 100 ng of random hexamers (Takara Bio, Otsu, Shiga, Japan), 10 mM dithiothreitol (Invitrogen, Carlsbad, CA, USA) and 0.5 mM deoxynucleotide triphosphates (dNTPs) (Takara Bio). After heating the solution to 60 °C for 5 min and incubating the mixture at 37 °C for 5 min, 200 U of recombinant murine leukemia virus reverse transcriptase (Invitrogen) was added and the mixture was incubated at 37 °C for 45 min. The reverse transcription was terminated at 95 °C for 5 min.

### 4.2. Selection of Primer Pairs for qPCR

Primers for qPCR were designed by Primer Express Software (Applied Biosystems, Foster City, CA, USA). PCR reactions (final volume 13 µL) contained 2× SYBR Green Master Mix (Brilliant II SYBR Green QPCR Master Mix, Agilent, Santa Clara, CA, USA), 2.7 ng of human cDNA or appropriate amount of the external standards (see below) and 2.5 pmol of primers for YKL-40. The PCR reactions were performed using Mx3005P QPCR System (Agilent) as follows: 10 min denaturation at 95 °C, 40 cycles of denaturation at 95 °C for 30 s, annealing at 55 °C for 1 min and polymerization at 72 °C for 1 min. Melting curves were generated after amplification. The PCR products were electrophoresed on a 10% polyacrylamide gel and analyzed using the Luminescent Image Analyzer (ImageQuant LAS 4000, GE Healthcare, Little Chalfont, UK). The primers’ sequences are listed in Figure S1. Chit1, AMCase, pepsinogen C, GAPDH and β-actin primers have been previously reported [45,46,47].

### 4.3. Construction of the Human Refs/YKL-40 Standard DNA

The human Refs/YKL-40 standard DNA (1,581 nucleotides; see Figure S2) was constructed using the following protocol: YKL-40 cDNA fragment covering the PCR target and flanking region was amplified from a human cDNA mixture as described above using the forward primer (Pst_YKL-40_Fw) containing PstI restriction site (at the 5' end) and the reverse primer (Quant_YKL-40_Rv) (Figure S4). The human Refs standard DNA [46], which consisted of AMCase/pepsinogen C/Chit1/GAPDH/β-actin, was PCR-amplified using Quant_Human_AMCase_Fw and PstI_Human_actin_Rv primers (Figure S4). Both PCR products were digested with PstI and ligated using T4 DNA ligase. The resulting fragments were amplified using Quant_Human_AMCase_Fw and Quant_YKL-40_Rv primers. PCR products were purified and cloned into the pGEM-T Easy vector. A plasmid containing the cDNA insert was selected and sequenced. The human Refs/YKL-40 standard DNA was prepared by PCR reamplification from the plasmid DNA using the same primers and the resulting PCR product was thereafter used as the human Refs/YKL-40 standard DNA (Figure S2).

### 4.4. Preparation of YKL-40 cDNAs Covering the Entire Coding Region

The cDNA covering the entire coding regions of YKL-40 were amplified from human tissue cDNAs by PCR using primers listed in Figure S5. The amplicons were subcloned into the pGEM-T Easy vector (Promega, Madison, WI, USA) with sequence verified (Figure S3). The subcloned fragments were reamplified from the plasmid DNAs using M13_Fw and M13_Rv (YKL-40) (Figure S5) and the resulting fragments were used as the region of YKL-40 open reading frame.

### 4.5. Standard Curves and mRNA Quantification Using Real-Time PCR

Standard qPCR was performed as follows: Initial denaturation and polymerase activation step at 95 °C for 10 min, 40 cycles of denaturation at 95 °C for 30 s, annealing at 55 °C for 30 s and polymerization at 72 °C for 10 s. The standard curves were constructed, and mRNA quantification was performed. Each sample was amplified in triplicate and each experiment was repeated at least two times.

## 5. Conclusions

Our study provides for the first time a comprehensive analysis of the relative expression levels of YKL-40 mRNA in comparison with mammalian chitinases in normal human tissues, although several total RNA samples were obtained only from a single individual. The data presented here can be considered as fundamentals for understanding the biological roles of YKL-40 and mammalian chitinases upon pathological changes in human tissues.

## Supplementary Materials

Supplementary materials can be found at http://www.mdpi.com/1422-0067/16/05/9922/s1.
