# Supplementary material for: Quantitative Real-Time PCR Analysis of YKL-40 and Its Comparison with Mammalian Chitinase mRNAs in Normal Human Tissues Using a Single Standard DNA
Source: Int J Mol Sci. 2015 Apr 30;16(5):9922–35. doi: 10.3390/ijms16059922 (PMC4463625; doi:10.3390/ijms16059922)
Supplement: Supplementary file 1 [file ijms-16-09922-s001.pdf]

Supplementary Information

YKL-40_Fw: GGCTTCTTCTGAGACTGGTGTTG
YKL-40_Rv: GAACCGGCCTGGAATTCC

Figure S1. The nucleotide sequences of the real-time PCR primers.

Mw 976864.4

CATGGAATTCTGGTCTGGGCCATTGATCTGGATGACTTCACTGGCACTTTCTGCAACCA
GGGCAAGTTTC**CCCTAATCTCCACCCTGAAGAA**GGCCCTCGGCCTGCAGAGTGC**AAGTT**
GCACGGCTCCAGCTCAGCCCATTGAGCCAATAACTGCTGCTCCCAGTGGCAGCGGGAAC
GGGAGCGGGAGTAGCAGCTCTGGAGGCAGCTCGGGAGGCAGTGGATTCTGTGCTGGCAG
AGCCAACGGCCTCTACCCCGTGGCAAATAACAGAAATGC***AGATCT***GCAGGCCACAGGGG
CCCAGGAGGATGAGTATGGACAGTTTCTCGTGAACTGTAACAGCATTCAGAATCTGCCC
AGCTTGACCTTCATCATCAATGGTGTGGAG**TTCCCTCTGCCACCTTCCT**CCTATATCCT
CAGTAACAACGGC**TACTGCACCGTGGGAGTCG**AGCCCACCTACCTGTCCTCCCAGAACG
GCCAGCCCCTGTGGATCCTCGGGGATGTCTTCCTCAGGTCCTACTATTCCGTCTACGAC
TTGGG***GTCGAC***TCTCTACCAGGAGTTCAATGGCCTGAAGAAGATGAATCCCAAGCTGAA
GACCCTGTTAGCCATCGGAGGCTGGAATTTCAGCACTCAGAAGTTCACAGATATGGTAG
CCACGGCCAACAACCGTCAGACCTTT**GTCAACTCGGCCATCAGGTT**TCTGCGCAAATAC
AGC**TTTGACGGCCTTGACCTTG**ACTGGGAGTACCCAGGAAGCCAGGGGAGCCCTGCCGT
AGACAAGGAGCGCTTCACAACCCTGGTACAGGACTTGGCCAATGCCTTCCAGCAG***CTCG***
AGGCCATCAATGACCCCTTCATTGACCTCAACTACATGGTTTACATGTTCCAATATGAT
TCCACCCATGGCAAATTCCATGGCACCGTCAAGGCTGAGAACGGGAAGCTTGTCATCA**A**
TGGAAATCCCATCACCATCTTCCAGGAGCGAGATCCCT**CCAAAATCAAGTGGGGCG**ATG
CTGGCGCTGAGTACGTCGTGGAGTCCACTGGCGTCTTCACCACCATGGAGAAGGCTGGG
GCTCATTTGCAGGGGGGAGCCAAAAGGGTCATCATCTCTGCCCCCTCTGCTGATGCCCC
CA***GCGGCCGC***ATGCAGAAGGAGATCACTGCCCTGGCACCCAGCACAATGAAGATCAAGA
TCATTGCTCCTCCTGAGCGCAAGTACTCCGTGTGGATCGGCGGCTCCATCCTGGCCTCG
CTGTCCACCTTCCAGCAGATG**TGGATCAGCAAGCAGGAGTATG**ACGAGTCCGGCCCCTC
C**ATCGTCCACCGCAAATGC**TTCTAGGCGGACTATGACTTAGTTGCGTTACACCCTTTCT
TGACAAAACCTAACTTGCGCAGAAAACAAGATGAGATTG***CTGCAG***TACATGTTGAGGCT
GGGGGCTCCTGCCAGTAAGCTGGTGATGGGCATCCCCACCTTCGGGAGGAGCTTCACTC
T**GGCTTCTTCTGAGACTGGTGTTG**GAGCCCCAATCTCAGGACCG**GGAATTCCAGGCCGG**
TTCACCAAGGAGGCAGGGACCCTTGCCTACTATGAGAGAATTCCATG

Figure S2. Nucleotide sequence of the human Refs/YKL-40 standard DNA. The human Refs/YKL-40 standard DNA (1581 nucleotides long) contained six cDNA fragments (shown in different colors) that covered the PCR target regions (shown in bold and underlined) and 44–143 nucleotides of the flanking regions and contained the Bg1II, SalI, XhoI, NotI and PstI restriction sites (shown in bold and italics).

MW 926,190.6

```
GTTTTCCCAGTCACGACGTTGTAAAACGACGGCCAGTGAATTGTAATACGACTCACTAT
AGGGCGAATTGGGCCCGACGTCGCATGCTCCCGGCCGCCATGGCGGCCGCGGGAATTCG
ATTCATGGGATCCCCTGTCTAGGTAGCTGGCACCAGGAGCCGTGGGCAAGGGAAGAGGC
CACACCCTGCCCTGCTCTGCTGCAGCCAGATGGGTGTGAAGGCGTCTCAAACAGGCTTT
GTGGTCCTGGTGCTGCTCCAGTGCTGCTCTGCATACAAACTGGTCTGCTACTACACCAG
CTGGTCCCAGTACCGGGAAGGCGATGGGAGCTGCTTCCCAGATGCCCTTGACCGCTTCC
TCTGTACCCACATCATCTACAGCTTTGCCAATATAAGCAACGATCACATCGACACCTGG
GAGTGGAATGATGTGACGCTCTACGGCATGCTCAACACACTCAAGAACAGGAACCCCAA
CCTGAAGACTCTCTTGTCTGTCGGAGGATGGAACTTTGGGTCTCAAAGATTTTCCAAGA
TAGCCTCCAACACCCAGAGTCGCCGGACTTTCATCAAGTCAGTACCGCCATTTCTGCGC
ACCCATGGCTTTGATGGGCTGGACCTTGCCTGGCTCTACCCTGGACGGAGAGACAAACA
GCATTTTACCACCCTAATCAAGGAAATGAAGGCCGAATTTATAAAGGAAGCCCAGCCAG
GGAAAAAGCAGCTCCTGCTCAGCGCAGCACTGTCTGCGGGGAAGGTCACCATTGACAGC
AGCTATGACATTGCCAAGATATCCCAACACCTGGATTTCATTAGCATCATGACCTACGA
TTTTCATGGAGCCTGGCGTGGGACCACAGGCCATCACAGTCCCCTGTTCCGAGGTCAGG
AGGATGCAAGTCCTGACAGATTCAGCAACACTGACTATGCTGTGGGGTACATGTTGAGG
CTGGGGGCTCCTGCCAGTAAGCTGGTGATGGGCATCCCCACCTTCGGGAGGAGCTTCAC
TCTGGCTTCTTCTGAGACTGGTGTTGGAGCCCCAATCTCAGGACCGGGAATTCCAGGCC
GGTTCACCAAGGAGGCAGGGACCCTTGCCTACTATGAGATCTGTGACTTCCTCCGCGGA
GCCACAGTCCATAGAATCCTCGGCCAGCAGGTCCCCTATGCCACCAAGGGCAACCAGTG
GGTAGGATACGACGACCAGGAAAGCGTCAAAAGCAAGGTGCAGTACCTGAAGGACAGGC
AGCTGGCGGGCGCCATGGTATGGGCCCTGGACCTGGATGACTTCCAGGGCTCCTTCTGC
GGCCAGGATCTGCGCTTCCCTCTCACCAATGCCATCAAGGATGCACTCGCTGCAACGGC
TCGAGGTCAAATCACTAGTGAATTCGCGGCCGCCTGCAGGTCGACCATATGGGAGAGCT
CCCAACGCGTTGGATGCATAGCTTGAGTATTCTATAGTGTCACCTAAATAGCTTGGCGT
AATCATGGTCATAGCTGTTTCCTG
```

Figure S3. Nucleotide sequence and calculated molecular weight of YKL-40 open reading frame. ATG start codon is highlighted in bold. Open reading frame of YKL-40 is shown underlined.

```
PstI_YKL-40_Fw: TGACCTGCAGTACATGTTGAGGCTGGGGGCTCCTG

Quant_YKL-40_Rv: CATGGAATTCTCTCATAGTAGGCAAGGGTCCCTGC

Quant_Human_AMCase_Fw: CATGGAATTCTGGTCTGGGCCATTGATCTGGATGA

PstI_Human_actin_Rv: TGACCTGCAGCAATCTCATCTTGTTTTCTGCGCAA
```

Figure S4. Forward and reverse primers used to construct the standard template DNA. The primers contains the PstI recognition sequence (underlined) and the 4 bases-long extra nucleotides (boldfaced) to efficiently cleave close to the end of the amplified cDNAs by PstI.

```
Entire YKL-40_Fw: CATGGGATCCCCTGTCTAGGTAGCTGGCACCAGGA
Entire YKL-40_Rv: TGACCTCGAGCCGTTGCAGCGAGTGCATCCTTGAT

M13_Fw: GTTTTCCCAGTCACGAC
M13_Rv: CAGGAAACAGCTATGAC
```

Figure S5. Primers for amplifying entire coding cDNAs by PCR.
